# Rotterdam Aphasia Therapy Study (RATS) – 3: “The efficacy of intensive cognitive-linguistic therapy in the acute stage of aphasia”; design of a randomised controlled trial

**DOI:** 10.1186/1745-6215-14-24

**Published:** 2013-01-23

**Authors:** Femke Nouwens, Diederik WJ Dippel, Marjolein de Jong-Hagelstein, Evy G Visch-Brink, Peter J Koudstaal, Lonneke M L de Lau

**Affiliations:** 1 Department of Neurology, Erasmus MC – University Medical Center Rotterdam, P.O. Box 2040, Rotterdam, CA, 3000, The Netherlands; 2 Department of Neurology, Slotervaart Hospital, Amsterdam, The Netherlands

**Keywords:** Aphasia, Stroke, Cognitive-linguistic therapy, Treatment, Timing, Intensity, RCT

## Abstract

**Background:**

Aphasia is a severely disabling condition occurring in 20 to 25% of stroke patients. Most patients with aphasia due to stroke receive speech and language therapy. Methodologically sound randomised controlled trials investigating the effect of specific interventions for patients with aphasia following stroke are scarce. The currently available evidence suggests that intensive speech and language therapy is beneficial for restoration of communication, but the optimal timing of treatment is as yet unclear.

In the Rotterdam Aphasia Therapy Study-3 we aim to test the hypothesis that patients with aphasia due to stroke benefit more from early intensive cognitive-linguistic therapy than from deferred regular language therapy.

**Methods/design:**

In a single blinded, multicentre, randomised controlled trial, 150 patients with first ever aphasia due to stroke will be randomised within two weeks after stroke to either early intensive cognitive-linguistic therapy (Group A) or deferred regular therapy (Group B). Group A will start as soon as possible, at the latest two weeks after stroke, with a four week period of one hour a day treatment with cognitive-linguistic therapy. In Group B professional speech and language therapy is deferred for four weeks. After this period, patients will follow the conventional procedure of speech and language therapy. Participants will be tested with an extensive linguistic test battery at four weeks, three months and six months after inclusion. Primary outcome measure is the difference in score between the two treatment groups on the Amsterdam-Nijmegen Everyday Language Test, a measure of everyday verbal communication, four weeks after randomisation.

**Trial registration:**

This trial is registered in the Dutch Trial Register (http://www.trialregister.nl), NTR3271.

## Background

About one fifth to a quarter of all stroke patients suffer from aphasia [1]. Aphasia after stroke is a major health problem with dramatic consequences for the quality of life of affected individuals. Communication is essential in daily life and may influence the outcome of rehabilitation [2], since different forms of therapy are usually instructed verbally. Hence, speech and language therapy (SLT) is considered very important in the acute phase after stroke.

The effectiveness of SLT has been evaluated in a variety of studies, many of which relied on small samples and were of limited methodological quality. Recently the Cochrane Collaboration has published a review of 39 trials on the efficacy of language therapy for aphasia after stroke [3]. The authors conclude that there is some evidence that SLT is more effective than no SLT for recovery of communication after stroke and that efficacy of SLT seems to be influenced by intensity of therapy. However, they emphasise that these results should be interpreted cautiously, as many studies lack proper methodology and comparison across studies is hampered by a large degree of heterogeneity regarding characteristics of the study population, applied treatment methods, timing and duration of therapy, and outcome assessments.

There are two main approaches in aphasia treatment: cognitive-linguistic therapy (CLT) and communicative or functional therapy [4]. CLT focuses on deficits in linguistic components, such as semantics (word meaning), phonology (speech sounds) and syntax (sentence level), and aims at restoring linguistic processes that are the foundation of language. Communicative therapy focuses on compensation by making use of all communicative channels; patients learn to utilise preserved verbal as well as nonverbal communicative functions. Communicative therapy is provided in a realistic everyday environment and uses gestures, communication aids, such as an icon board, role plays and the Promoting Aphasics’ Communicative Effectiveness (PACE) method [5]. CLT is mostly applied in early stages after stroke and communicative therapy later on.

Our group previously studied the efficacy of CLT, aimed at semantic and phonological processing, in comparison to communicative therapy in the acute stage of aphasia in the Rotterdam Aphasia Therapy Study (RATS) – 2 [6]. A total of 80 aphasic patients were randomised to either CLT or communicative therapy for six months, starting within three weeks after the stroke. We found no significant difference between groups on the primary outcome measure, the Amsterdam-Nijmegen Everyday Language Test, A-scale (ANELT-A) [7]. However, the majority of the secondary outcome measures on semantics and phonology were in favour of CLT. Perhaps the treatment intensity of both interventions, on average 2.1 hours per week, was insufficient to generate a significant treatment effect on top of spontaneous recovery [8-10].

Recovery of communication usually occurs shortly after stroke [11-14]. Most likely restoration of the perilesional network in the left hemisphere is the primary mechanism underlying this spontaneous recovery [15]. Therapeutic strategies to restore cerebral blood flow, such as thrombolysis, enhance spontaneous recovery. SLT is aimed at stimulating cortical networks involved in language, hence increasing blood flow to these damaged areas. CLT especially stimulates the linguistic cortical network through specific exercises for linguistic components, such as semantics, phonology and syntax [4]. Hence SLT, and in particular CLT, is thought to contribute positively to spontaneous recovery of language [14,15]. Some, therefore, claim that the focus of SL-therapists in the acute stage of aphasia, when restoration of the linguistic network is still plausible, should be on CLT [14,16].

In this respect, several clinical studies suggest that therapy provided immediately after stroke results in more beneficial effects than deferred treatment [11]. In a review article that was not restricted to randomised trials but also included studies with other designs, the authors conclude that SLT in the acute stages of aphasia following stroke is almost twice as effective as natural recovery alone [10]. This assumes the presence of a “critical period” after stroke during which the brain is more susceptible to rehabilitation. Furthermore, it implies that SLT should be initiated as soon as possible after stroke. This assumption also suggests that if SLT is initiated too late, recovery might be restricted. The length of this supposed “critical period”, however, is unclear and optimal timing of therapy remains uncertain.

A second mechanism of recovery is neural plasticity [17]. Intensive training, for instance, massed practise, is thought to trigger remodelling and consolidation of neural networks [15]. Efficacy of SLT is considered to be related to intensity [8]. In the Cochrane systematic review of randomised controlled trials on SLT for aphasic stroke patients, it was shown that efficacy of SLT positively correlated with treatment intensity, although this was related to more therapy drop-outs [3]. However, a recently published pilot study on intensive SLT in 59 acute stroke patients with aphasia suggests that early intensive SLT is feasible in the acute stage after stroke [18]. The number of drop-outs or deaths reported in the intervention group with daily therapy was not higher than in the control group with usual care therapy.

Another trial, in which 123 aphasic patients were randomised for intensive SLT (three weeks of daily SLT for 45 minutes on workdays, starting within two days after stroke) or control condition (no SLT for three weeks) in the acute stage of aphasia, showed no significant differences between groups on the primary outcome measure ANELT-A [7,19]. The authors conclude that not all patients with aphasia after stroke benefit from early intensive SLT, but it can be questioned whether therapy in this study was sufficiently intensive [8-10].

Based on the currently available evidence, we suggest an optimal regime of early initiated intensive CLT for aphasia after stroke. This regime will be studied in the Rotterdam Aphasia Therapy Study (RATS) – 3.

### Objective

The objective of this study is to test the hypothesis that patients with aphasia after stroke benefit more from early initiated intensive cognitive-linguistic therapy than from deferred regular SLT.

## Methods

**Figure 1:**
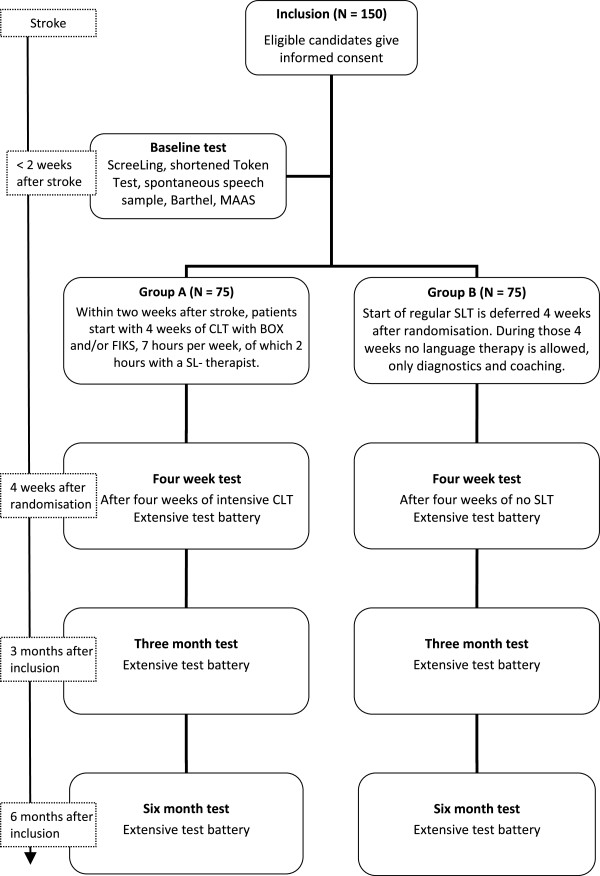
Flow diagram of the RATS-3 study design.

RATS-3 is a multicentre, stratified (for centre of inclusion and severity of aphasia) single-blinded randomised controlled trial with parallel groups (see Figure 1).

### Participants and recruitment

RATS-3 is coordinated by the Erasmus MC – University Medical Center Rotterdam, and over 40 hospitals, nursing homes and rehabilitation centres in The Netherlands participate. SL-therapists in participating centres are trained and supervised by the trial team.

Hospitalised patients with aphasia due to stroke are screened by the local SL-therapist for eligibility with the inclusion and exclusion criteria within two weeks after stroke (see Table 1).

**Table 1:** Eligibility criteria for the Rotterdam Aphasia Therapy Study (RATS) – 3

**Inclusion criteria:**			
1. Aphasia after stroke, determined by a neurologist or rehabilitation physician and speech and language therapist;			
2. Within two weeks after stroke;			
3. Testable with ScreeLing [20];			
4. Aphasia ascertained with shortened Token Test [21] and/or a score <5 on Goodglass Aphasia Severity Rating Scale [22];			
5. Age between 18 and 85 years;			
6. Language near native Dutch;			
7. Life expectancy of more than six months.			
**Exclusion criteria:**			
1. Pre-existing aphasia;			
2. Subarachnoid/subdural haemorrhage/hematoma;			
3. Severe threats to success and/or feasibility of language therapy:			
a. Severe dysarthria;			
b. Premorbid dementia;			
c. Illiteracy;			
d. Severe developmental dyslexia;			
e. Severe visual perceptual disorders;			
f. Recent psychiatric history.			

Written informed consent is acquired by the local SL-therapist from eligible patients and/or their family. Patient information and consent forms are approved by the medical ethical committee of the Erasmus MC – University Medical Center Rotterdam. Local SL-therapists will inform the RATS-3 team of every new participant.

### Baseline tests

All candidates will be tested with the ScreeLing [20], a screening instrument for aphasia. Aphasia is ascertained by the shortened version of the Token Test [21] and a sample of spontaneous speech assessed with the Goodglass Aphasia Severity Rating Scale [22]. Activities of daily life will be reported with the Barthel Index [23] and observational data on social and (neuro)psychological functioning and wellbeing will be collected with the MAAS (Multi-axial Aphasia System [24]).

### Randomisation

Each participant is assigned to either Group A or Group B by restricted randomisation via stratification for severity of aphasia and centre of inclusion. The allocation sequence is computer generated and concealed in consecutively numbered, opaque, sealed envelopes. The trial coordinator randomises participants to treatment groups after severity of aphasia is assessed. A score on the Goodglass Aphasia Severity Rating Scale [22] of 0 to 2 is considered to reflect severe aphasia and a score of 3 to 5 reflects moderate to mild aphasia.

### Intervention

As soon as possible after randomisation the intervention period of four weeks starts, during which Group A receives early intensive CLT and Group B receives no SLT.

### Intervention group: Group A (N = 75)

Participants allocated to Group A receive intensive CLT with the treatment programs BOX [25] and/or FIKS [26]. The BOX and FIKS programs are commonly used in The Netherlands and aim at the improvement of word finding (see Table 2). BOX focuses on semantics and FIKS on phonology. Both interventions are well outlined, which ensures homogeneity of treatment in this group [6]. BOX and FIKS consist of several subparts that provide a large number of specific exercises, to treat various layers of semantic and phonological processing. Exercises can be presented visually and/or orally and require receptive and productive skills. Each subpart contains different levels of complexity, which makes these programs suitable to all types and severity levels of aphasia. Both programs are also available on computer (eBOX and eFIKS) to facilitate homework.

**Table 2:** Illustration of CLT with the semantic therapy program BOX and the phonological program FIKS

**Semantic therapy program BOX **Subject: word meaning Objective: consolidate the internal semantic network to improve word finding Presentation: oral, visual or by computer (eBOX)				
**Examples**:				
Word level	Sentence level			
*Which word does not match?*	*Is this sentence correct?*			
Ruler	The balloon flies in the air.			
Musical scale	Correct.			
Gauge				
Balance	The candle is burning embittered.			
Measuring tape	Incorrect. *Please correct the sentence.*			
**Phonological therapy program FIKS** Subject: processing and production of speech sounds Objective: consolidate the internal phonological network and improve production of speech, to improve word finding Presentation: oral, visual or by computer (eFIKS)				
**Examples:**				
Word level				
*Which word is printed here?*	tion	trans	la	= translation
*Read it out loud please.*	ment	ta	tes	= testament
Sentence level				
*Please finish the sentence with a rhyming word*:		The enthusiastic amateur cook,		
		read the recipe carefully in his cooking- …		

Therapy will start at the latest two weeks after stroke. However, as soon as participants are included and randomised, therapy can be started.

Recent findings on intensity of treatment [3,8] suggest that one hour of language therapy per day is sufficiently intensive to generate an effect of therapy on top of the effect of spontaneous recovery. This high intensity is uncommon in The Netherlands. Therefore SL-therapists will treat participants at least two hours a week, supplemented with homework using paper or digital versions of the therapy programs. The SL-therapists register all therapy sessions in minutes on special registration forms. These forms will be handed to the patient and/or his caretaker also for homework registration.

The trial coordinator contacts SL-therapists every week to check whether the allocated treatment is adequately applied and ask if any problems arise complying with the protocol.

### Control group: Group B (N = 75)

Language therapy is deferred in Group B. Regular language therapy will start four weeks after randomisation. During these four weeks no SLT is allowed. SL-therapists, however, will be attentive to participants in Group B. They may inform the patient and his caretakers about aphasia and its consequences and provide advice to avoid severe communication distress. Additional diagnostic tests and specific observations on communicative functioning may be performed to set detailed therapy goals.

Therapy after four weeks by a SL-therapist will be arranged if the patient is discharged home. The trial coordinator will keep in contact with the patient during these four weeks. If the patient is released to a rehabilitation centre or nursing home, the coordinator will contact the SL-therapist after two weeks to evaluate whether the protocol can be followed correctly.

### Follow-up measurements

Verbal communicative abilities of participants will be evaluated four weeks after randomisation, three months after inclusion and six months after inclusion, using an extensive linguistic test battery (see Table 3). Tests requiring a verbal response are recorded digitally. All SL-therapists receive a manual for the administration of the linguistic tests. Results will be scored in a booklet containing all score forms per test moment. The trial team will score all tests and report the results to the SL-therapists.

**Table 3:** Linguistic test battery of RATS-3

**General communication tests**	
- ANELT-A: communicative functioning in daily life [7];	
- Semi-standardised interview for spontaneous speech rated with Goodglass Aphasia Severity Rating Scale [22];	
- Sabadel: connected speech [27];	
- ScreeLing: screening of three linguistic components: semantics, phonology and syntax [28];	
- Token Test, short version: measures severity of aphasia [21];	
- Boston Naming Test: identifies word finding difficulties [29].	
**Specific semantic tests**	
- Semantic Association Test, verbal version (SAT) [30];	
- Comprehensive Aphasia Test, word comprehension (CAT) [31];	
- Semantic Word Fluency [32].	
**Specific phonological tests**	
- Nonword repetition, PALPA [33];	
- Auditory Lexical Decision, PALPA [33];	
- Letter Fluency [34].	
**General**	
- Barthel Index: activities of daily life [23];	
- Multi-Axial Aphasia System (MAAS) [24];	
- Partner ANELT: partner’s perspective on the patient’s communicative functioning [35];	
- Self evaluation of communicative functioning on a 0 to10 scale;	
- EuroQol: quality of life [36];	
- Modified Rankin Scale: activities of daily life [37].	

Aphasia type will be determined with the Aachen Aphasia Test (AAT, [38]) between the fourweek and threemonth test. This period after spontaneous recovery is chosen because we assume aphasia type will then be stabilised.

### Sequel after the four week test

Regular language therapy will start in Group B and Group A continues with regular therapy after the fourweek test. Regular therapy in The Netherlands comprises a combination of CLT and communicative therapy and focuses mainly on therapy goals set by the patient. Regular therapy intensity is on average approximately two hours per week [39]. Registration of therapy sessions and therapy type (either CLT or communicative or a combination) will be continued, although not as meticulously as during the four weeks of intervention.

### Primary outcome

The primary outcome measure in RATS-3 is the difference in the score on the ANELT-A [7] at the four week test moment (after intervention) between the two groups. CLT aims at improving linguistic skills, which theoretically results in better daily communication. The A-scale of the valid and reliable ANELT [40] measures verbal communicative ability. Participants’ verbal responses to 10 everyday communicative scenarios are scored on a five-point scale for information content.

### Secondary outcomes

The difference in scores between groups at the four week test on the Semantic Association Test (SAT), verbal version [30]; Semantic Word Fluency [32], Psycholinguistic Assessment of Language Processing in Aphasia (PALPA), Nonword repetition [33]; PALPA, Auditory Lexical Decision [33]; and Letter Fluency [34] will be used as secondary outcome measures. Other secondary outcomes are differences in all test scores at three months, and differences in scores on the EuroQol (quality of life) [36] and Modified Rankin Scale (functional outcome) [37].

### Tertiary outcomes

Scores on the above mentioned tests at six months after inclusion, including ANELT-A, will be used as tertiary outcome measures.

### Sample size

A sample of 75 participants in each group, a total of 150 participants, is estimated to provide 84% power to detect a statistically significant difference on the primary outcome measure between groups at a 5% two-sided significance level. An inclusion period of two years is estimated to be required for recruitment.

### Blinding

Due to the intervention type, therapy or no therapy, it is impossible for participants and SL-therapists to be blinded for intervention. Assessment of the primary outcome, however, will be blinded. Two experienced independent observers, who are blinded for treatment allocation and test moment, will score the primary outcome measure ANELT-A. The mean score of both independent observers will be used in the analyses. Interobserver agreement will be assessed by means of a plot of differences between scores versus their mean. The mean difference between observers will be calculated with a 95% confidence interval.

### Statistical analyses

Difference in score on ANELT-A between groups will be compared with analysis of covariance (ANCOVA) with a 95% confidence interval, adjusted for baseline severity. Baseline severity is determined according to the Goodglass Aphasia Severity Rating Scale [22] in a sample of spontaneous speech.

This method will also be used for the additional linguistic tests in the secondary and tertiary outcome measures.

#### Ethics

The RATS-3 study protocol is approved by the independent medical ethical committee of the Erasmus MC – University Medical Center Rotterdam (MEC-2005-347), and registered in the Dutch Trial Register (NTR3271) [41].

### Trial status

The trial started January 2012. We estimate that inclusion will be finished in January 2014.

## Appendix

In Table 4, we publish the list of participating centres updated until August 2012. This list is not final as we are still actively recruiting centres.

**Table 4:** Provisional list of participating centres and the principal local investigators*

**Hospitals**	**Principal Investigator**
Haven Ziekenhuis, Rotterdam	Ida Boas
Sint Franciscus Gasthuis, Rotterdam	Joyce van Dalen
Ikazia Ziekenhuis, Rotterdam	Mathanja Sibon
Maasstad Ziekenhuis, Rotterdam	Fabiënne Stok
Vlietland Ziekenhuis, Schiedam	Obbe de Roos
IJsselland Ziekenhuis, Capelle aan de IJssel	Ingrid Arp
Reinier de Graaf Gasthuis, Delft	Jolanda van Veldhuizen
MCH Westeinde, Den Haag	Marike Kamphuis,
MCH Antoniushove, Leidschendam	Christa Kerkhof
Haga Ziekenhuis, Den Haag	Nienke Splinter
VUMC, Amsterdam	Antoinette Keulen
Diaconessenhuis, Meppel	Cock Meijs
Rivas, Beatrix Ziekenhuis, Gorinchem	Wieteke Musterd
Amphia Ziekenhuis, Breda	Sylvia Goosen
**Rehabilitation centres**	**Principal investigator**
Laurens Antonius, Binnenweg, Rotterdam	Siri Siepel
Laurens Antonius, IJsselmonde, Rotterdam	Ankerien Gerritse
Rijndam, Central clinic, Rotterdam	Mieke van de Sandt
Rijndam, Central outpatient centre, Rotterdam	Miranda de Waard-van Rijn
Rijndam, Vlietland outpatient centre, Schiedam	Merle Paterson
Rijndam, De Waarden outpatient centre, Gorinchem	Wieteke Musterd
Vlietland Ziekenhuis outpatient centre, Schiedam	Obbe de Roos
Centrum voor Reuma en Revalidatie, Rotterdam	Anke de Meij
Maasstad Ziekenhuis outpatient centre, Rotterdam	Fabiënne Stok
Zonnehuis, Vlaardingen	Suzanne van Almenkerk
Sophia Revalidatie, Delft	Marjolein Zomerdijk
Stichting Pieter van Foreest, Delft	Margot van Vorstenbosch
Sophia Revalidatie, Den Haag	Elske van Egmond
Florence, Gulden Huis, Den Haag	Charlotte Schmitz
Florence, Huize Westhoff, Rijswijk	Charlotte Schmitz
Florence, Mariahoeve, Den Haag	Charlotte Schmitz
Zonnehuis, Amstelveen	Jan van Olsthoorn
Reade, Amsterdam	Laurien Sietsma
De Volckaert-SBO, Oosterhout	Marianne Slabbekoorn
Stichting Elisabeth, Breda	Judith van Bree
Thebe Aeneas, Breda	Cirsten van Gelder
De Riethorst-Stromenland, Geertruidenberg	Cirsten van Gelder
Stichting Bilthuysen, De Biltse Hof, Bilthoven	Melanie Swens
Zorgcombinatie Noorderboog, Reggersoord, Meppel	Nelleke Loseman
Stichting Groenhuysen, Roosendaal	Saskia Aarts
Avoord Zorg en Wonen, Etten-Leur	Nancy Schuurman
Stichting SHDH, Janskliniek, Haarlem	Annet Voogd
Stichting Afasietherapie, Amsterdam	Marieta Gerarts
Rivas, Waerthove, Sliedrecht	Wieteke Musterd
Rivas, Lingesteijn, Leerdam	Wieteke Musterd
Rivas, Het Gasthuis, Gorinchem	Wieteke Musterd

## Abbreviations

ANELT: Amsterdam-Nijmegen everyday language test; CAT: Comprehensive aphasia test; CLT: Cognitive linguistic treatment; MAAS: Multi-axial aphasia system; PACE: Promoting Aphasics’ Communicative Effectiveness; PALPA: Psycholinguistic assessment of language production in aphasia; RATS-3: Rotterdam aphasia therapy study – 3; SAT: Semantic association test; SLT: Speech and language therapy; SL-therapist: Speech and language therapist.

## Competing interests

The authors declare that they have no competing interests.

## Authors’ contributions

DD, PK, MJ, EV and LL conceived the study and designed this trial. LL is the principal investigator and FN is the trial coordinator. EV, MJ and FN will acquire the data, which FN will primarily analyse and interpret. LL and FN wrote the first draft of the manuscript, which was critically revised by MJ, EV, DD and PK. All authors have read and approved the final manuscript.

## Authors’ information

MJ, DD, PK and EV conducted the preceding RATS-2 study in which the efficacy of impairment directed language therapy was tested in comparison to communicative therapy [6]. MJ initiated RATS-3 and started data collection. From 1 January 2012, FN has taken over the coordinating tasks of MJ.
